# Developing and designing an internet-based support and education program for patients awaiting kidney transplantation with deceased donors through: a Delphi study

**DOI:** 10.1186/s12882-023-03364-2

**Published:** 2023-10-25

**Authors:** Kristina Nilsson, Gerhard Andersson, Peter Johansson, Johan Lundgren

**Affiliations:** 1 Department of Health, Medicine and Caring Sciences, Linköping University, Norrköping, Sweden; 2 Department of Internal Medicine and Department of Health, Medicine and Caring Sciences, Linköping University, Norrköping, Sweden; 3 Department of Behavioural Sciences and Learning, Department of Biomedical and Clinical Sciences, Linköping University, Linköping, Sweden; 4 Department of Clinical Neuroscience, Karolinska Institute, Stockholm, Sweden

**Keywords:** Kidney transplantation, Education, Psychological support, Internet-based intervention, Consensus development

## Abstract

**Aims:**

The aim of this study was to develop and refine the content and design of an internet-based support and education program for patients awaiting kidney transplantation from deceased donors.

**Design:**

A Delphi process was used.

**Methods:**

A prototype internet-based intervention was drafted, based on previous research. The intervention included educational and psychological support to manage the uncertain waiting time and specific education enabling preparation for transplantation and adjustment to life after transplantation. In a two-round Delphi process, patients who had received a kidney transplant from a deceased donor within the last 2 years (*n* = 27), significant others (*n* = 6), health-care personnel with renal (*n* = 20) or transplant (*n* = 14) expertise, rated importance of content and design aspects of the prototype intervention on a 5-point scale using web questionnaires. A median of ≥ 3 was considered as consensus. Quantitative data was analyzed using descriptive statistics. Free text answers were encouraged and analyzed using deductive content analysis. The STROBE-checklist was used.

**Results:**

Consensus was reached for all suggested content and design items in round 1, with median ratings of 4 or 5. Qualitative analysis from round 1 suggested four new content and design items which were rated in round 2, on all which consensus was reached; information about life with young children as relatives, expansion of kidney transplantation specific information, program extension by one week and individualization by making information available based on individual needs.

**Conclusion:**

There was consensus among heterogenous experts regarding suggested educational and psychological support content and design aspects, and additional content and design aspects were identified for an internet-based support and education program for patients awaiting kidney transplantation from deceased donors.

## What does this paper contribute to the wider global community?

- This paper suggests content for future internet-based education and support programs for patients awaiting kidney transplantation from deceased donors.
- Important design aspects for such a program are discussed.
- It is important to consider different perspectives when preparing intervention content.

## Introduction

Patients awaiting kidney transplantation from deceased donors are at greater risk of experiencing physical and psychological health problems compared to those who have a living donor [1]. The waiting time is often long and dialysis treatment is often required during the waiting period. Hence, these patients do not only experience uncertain waiting times, but are also during dialysis profoundly affected physically and psychologically by their disease and treatment [1–3]. It has been suggested that implementation of interventions including systematic education and psychosocial support is needed to address these patients’ complex challenges [2, 3]. Recent studies have found pretransplant misconceptions among transplant candidates and shown that pretransplant expectations strongly influence satisfaction after transplantation [1, 3, 4]. This suggests that support interventions should be provided prior to transplantation and address the entire transplantation process, including assessment, waiting time and life after transplantation. In a qualitative study we found that patients, despite suggesting support in the form of group meetings during the wait for kidney transplantation, described not having energy to attend such meetings [3]. As these patients are limited by their disease and treatment as well as geographically dispersed, an internet-based education and support program may be a potential solution and provide more readily available and equal care. To our knowledge no systematic and standardized internet-based support and education program has yet been developed and tested for these patients.

## Background

An internet-based support and education program can be considered a complex intervention due to the number and flexibility of components involved, the range of behaviors targeted, the expertise required by the persons delivering the intervention as well as the context in which it is intended to be used [5]. In accordance with the Medical Research Council’s (MRC) framework for developing complex interventions [5], we have in a first phase drafted a potential intervention to answer to the needs of patients awaiting kidney transplantation from deceased donors, based on previous literature [1, 4] and our previous interview study [3]. The intervention includes educational and psychological support to manage the uncertain waiting time as well as specific education to enable preparation for transplantation and adjustment to life after transplantation.

In a second phase we developed a prototype guided internet-based support and education program for the patient group. Detailed educational content was developed from interview data, previous research and information leaflets produced by the Swedish transplantation centers. The support aspects of the program were inspired by previous cognitive behavioral therapy (CBT) programs used for patients with medical conditions [6], using acceptance, problem solving and behavioral activation. CBT, a type of psychotherapy based on the concept of changing unhelpful or unhealthy thoughts, feelings and behaviors [7] as well as Acceptance and commitment therapy (ACT) [8], which targets the ability to lead a meaningful life also in the presence of unwanted inner experiences, have been used in patients with chronic somatic conditions to reduce psychological and somatic problems [9, 10]. They have also been used to support patients with somatic disease in managing their symptoms, treatment and self-care [11–13]. Importantly, CBT and internet-delivered cognitive behavioral therapy (ICBT) are also promising for prevention of depressive disorders and anxiety in individuals who do not have but are at increased risk of developing these disorders [14, 15]. In a third phase we intended to refine the prototype intervention regarding content and key uncertainties through involvement by stakeholders, in accordance with the MRC/NIHR framework [5] before evaluation through a feasibility study.

### Aims

This study aimed to further develop and refine the content and design of an internet-based support and education program for patients awaiting kidney transplantation from deceased donors, by involving important stakeholders in a Delphi study.

## Method

### The program prototype

The program prototype consisted of eight modules, spanning over eight weeks. Each module comprised text, illustrations, short videos, a prompt for physical activity and weekly homework assignments, on which feedback was to be given each week by a support person. Modules adapted for participants who are transplanted during the course of the program were available. Table 1 shows an overview of the program prototype.

**Table 1:** Overview of the support and education program prototype

Week	Content	Aims and objectives
1	Introduction including goal setting	To inform and engage, and to set individual goals for the program
2	Education regarding kidney failure, treatment options, living with kidney failure, kidney transplantation specific information including medications, lifestyle habits and complications	To learn more about kidney failure, treatments, and what it may be like to live with renal failure while waiting for or after kidney transplantation
3	Acceptance	To understand how acceptance of unwanted experiences may facilitate engaging in meaningful activities, and to identify life values which may guide own behavior
4	Problem solving	To identify aspects which are perceived as problematic and practice ways to solve or relate to these aspects
5	Behavioral activation I	To become aware of how behavior, thoughts and wellbeing are connected and to map own behavior
6	Behavioral activation II	To identify desired change and practice reducing negative behavior and increasing positive behavior
7	Behavioral activation III	To identify desired change and practice reducing negative behavior and increasing positive behavior
8	Summary and consolidating skills learned	To summarize skills learned and maintain implemented changes

### Design and setting

**Fig. 1:**
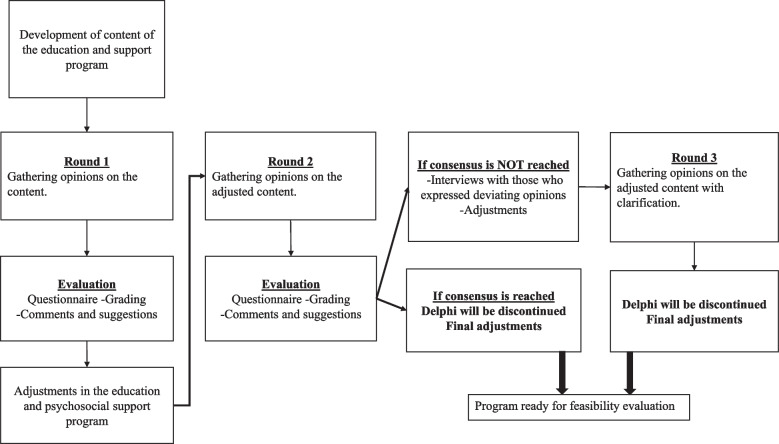
Overview of Delphi-plan for this study

The study followed a Delphi process, involving several groups of stakeholders as experts; patients, their significant others and health care professionals with knowledge and experience within renal and transplant care. The Delphi method is an iterative multistage process, appropriate for engaging a large number of experts (referred to as participants in this paper) in a systematic process of reaching consensus on a topic where the required information is limited or contradictory [16]. The most important characteristics of a Delphi study are iteration, anonymity between experts, providing participants with feedback of group ratings, allowing participants to give and, after receiving feedback, change their opinion freely [16, 17]. To facilitate these characteristics web-questionnaires were used, hence avoiding physical meetings where one or more of the participants may dominate the consensus process, ensuring anonymity between participants [16, 17], and enabling inclusion of a large number of participants who were geographically dispersed. The questionnaire and feedback process is to be repeated until consensus is reached, or a predetermined number of rounds are completed [16]. We anticipated that some questions would not reach consensus after two rounds, and therefore modified the Delphi process [18] by planning to perform telephone interviews with those who expressed dissenting opinions to clarify the reasons for disagreements, make adjustments to the program before performing a third and final round, as shown in Fig. 1. The Strengthening the reporting of observational studies in epidemiology (STROBE) checklist was used [19].

### Sampling and recruitment

It has been recommended that participants in Delphi method studies should qualify for selection because they are representative of their profession or are unlikely to be challenged as experts in the field. Credibility and acceptance of quality indicators are enhanced if the expert panel is heterogenous, reflecting the full range of stakeholders [18]. In order to capture divergent experiences and opinions about the support and education program the participants therefore included four groups of stakeholders; patients who had received a kidney transplant from a deceased donor within the last 2 years after some time on a waiting list, significant others’ of these patients, health-care personnel in the care of patients awaiting kidney transplantation from deceased donors and kidney transplant specialist personnel.

Recruitment for the Delphi study started in November 2021 and was open until the beginning of April 2022. Clinical managers of renal units and transplant centers across Sweden were approached via e-mail with study information and asked for approval to recruit patients and healthcare personnel as above. However, despite reminders very few gave their approval. Those who declined referred to high workload in times of the Covid-19 pandemic. Hence recruitment was expanded to two Swedish kidney/transplantation patient interest groups in social media, and for health-care personnel e-mail with information about the study was sent out by leads of four renal/transplantation interest organizations: Swedish transplantation society, Swedish nephrology association, Swedish nephrology nurses’ association and one transplant nurses’ network. By doing so we enabled participation of all groups of experts from all parts of Sweden. Those who wished to participate registered on the study website by registering their e-mail address. Written informed consent was collected at the time of registration. They then received an individualized link to the study, sent to their e-mail address, which ensured that the e-mail address was correct. The participants were instructed to follow the link to reach the questionnaire. For patients recruited through renal clinics, study information letters including how to find the study website were sent by post. Those who wished to participate registered as above. Participating patients were asked to leave their own phone number on the study website if they had a significant other willing to participate and whom they allowed us to contact. Those patients were called, and provided the phone number of their significant other, who was called and informed about the study, and upon approval sent a link to the study website where they could read study information and register for participation.

Four of the patients who had registered for participation in study after finding the information in social media did not fit the inclusion criteria, as they had been transplanted 9–192 months longer than prescribed for the study. As they contributed valuable data which did not alter the outcomes we included them in the study.

### Procedure and data collection

The participants were asked to complete two to three rounds of questionnaires focusing on their views on the education and support program content and design and possible interview. Responses to each round of questionnaires were analyzed and summarized and returned to the participants with a new questionnaire. This process was predetermined to be repeated at least twice [16–18], possibly followed by interviews and a third round. However, these last two steps were not necessary. Data were collected using a study web-platform, which had previously been extensively used and tested.

Once registered, participants were able to view the study page with information about the study and the support and education program, and to start answering the questionnaire. Background data of all participants were collected including age and gender, for health-care professionals also profession, specialty and work experience, for patients marital status, level of education, how long they had waited for a kidney transplant and time since transplantation, what form of dialysis (if any) the patient had while waiting for transplantation, and for significant others type of relation to the transplanted person.

For the first round, the support and education program prototype and the contents were described in text, pictures and through an introductory film to guide participants through the suggested program platform on the study web page. The questionnaire was developed to match the different chapters and content of the program and also stated questions regarding design. The questionnaire had been pilot tested by two patients and two health-care personnel, after which minor changes were made for clarification.

There are no strict rules for establishing when consensus is reached [16, 17]. However, the level or type of consensus should be defined in advance. Different criteria for describing when consensus is reached have been described. Among them is the criterion selected for this study. In the first round participants were asked to complete the questionnaire evaluating the different topics and content, structure and design of the program with ratings on a scale 1–5, where 1 means low importance and 5 very important. The topics that receive a median rating of 3.0 or greater were accepted as consensus [17]. Participants were also encouraged to leave written comments and suggestions regarding each topic. As ICBT involving therapeutic support are associated with better effects than unguided ICBT [9], participants were also asked what qualifications such a support-person for the program in question should have. Anticipated time required for round 1 was 30–40 min. Recruitment and participation in round 1 was consecutive. Round 1 closed for participation 22 weeks after first inclusion and participants were sent up to three reminders.

In the second round the participants received feedback with quantitative group results (median ratings) of each item as well as qualitative feedback such as abstracts of participants´ comments. Comments and suggestions from the different participants were kept anonymous for the participants in order to ensure private decision making. Suggestions made in round 1 regarding topics that could be expanded on in the program were described in round 2, and participants were asked to rate the importance of these suggestions on the same scale 1–5 as used in round 1. For round 2, participants were given 8 weeks to respond.

### Analysis

Quantitative analysis, using IBM SPSS Statistics version 28.0, was presented as median and quartiles for each item in the questionnaire. Differences between groups regarding ratings of all items were analyzed, to see if patients and significant others jointly had rated differently than healthcare personnel. Mann Whitney´s test was performed as we compared two independent groups which were not normally distributed, and the variable was ordinal data.

Qualitative data from questionnaires were analyzed according to the principles of a deductive content analysis. Prior to beginning analysis, key concepts were developed as initial coding categories in relation to how questions were asked in the questionnaire. Main categories were program content, program layout and design and implementation aspects. The first author read all qualitative data twice to familiarize herself with the data and its context. Whenever there was uncertainty of the meaning of the text, the author reviewed the questionnaire as a whole for better understanding and to ensure that the data was analyzed in the correct context. All text that on first impression appeared to answer the aim was highlighted. In the next step all highlighted passages were coded using the predetermined codes. Any text that could not be categorized with the initial coding scheme was given a new code. NVIVO software was used to ensure that codes could be traced to the setting they were collected [20].

## Results

### Round 1

#### Participants

**Fig. 2:**
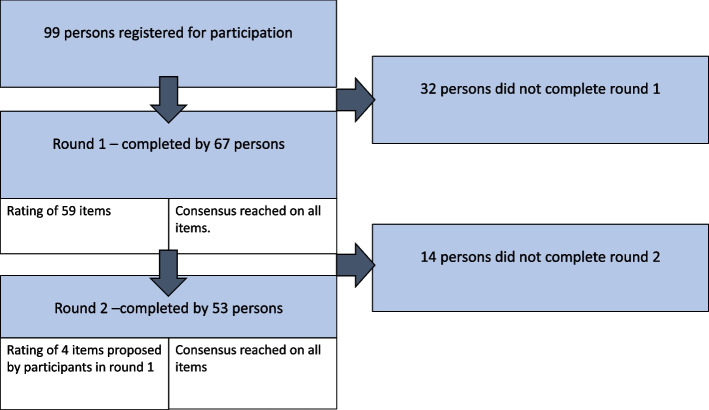
Flow diagram illustrating the two survey rounds and retention of participants between rounds of the Delphi study

A total of 99 individuals registered for participation, and 67 of these completed round 1, see Fig. 2. Persons who registered to participate in the study but did not complete round 1 (*n* = 32) and those who did complete the first round (*n* = 67) did not significantly differ regarding mean age (51 vs. 51, *p* = 0.89) and gender (females 66% vs. 73%, *p* = 0.45).

The characteristics of participants in round 1 are shown in Tables 2 and 3. Participants in the patient group were 48 per cent female, while among significant others 83 per cent were female. Mean age among patients was 57 years (SD ± 11) and among significant others 55 years (SD ± 20). The majority (82%) of participating patients were married or co-habiting. The participating significant others were either spouse or parent of the transplanted person. Median time patients had waited for transplantation was 7 months (min 1 and max 72 months). One quarter of patients had not required dialysis during their wait for transplantation. Patients and significant others were not asked about any prior transplantation or kidney donation experience. A total of 41 per cent and 50 per cent of participating patients and significant others respectively had university education.

**Table 2:** Participant characteristics; patients and significant others

	**Patients (*n* = 27)**	**Significant others (*n* = 6)**
Age year, Mean (SD)	57 (11)	55 (20)
Gender, female n (%)	13 (48)	5 (83)
**Marital status, n (%)**		
Married/co-habiting	22 (82)	N/A
Partner not cohabiting	2 (7)	N/A
Single	3 (11)	N/A
**Relationship to transplantee, n (%)**		
Spouse	N/A	5 (83)
Partner not co-habiting	N/A	0
Ex-partner	N/A	0
Parent	N/A	1 (17)
Child	N/A	0
Sibling	N/A	0
Friend	N/A	0
Other	N/A	0
**Educational level, n (%)**		
Elementary school	2 (7)	0
Upper secondary school	10 (37)	2 (33)
Vocational education	4 (15)	1 (17)
University	11 (41)	3 (50)
Time since tx, months, Md, Min/Max	14, 4/216	17, 6/22
Waiting time for tx, months, Md, Min/Max	7, 1/72	9, 3/72
**Type of dialysis, n (%)**		
None	7 (26)	3 (50)
HD in hospital	7 (26)	0
HD at home	1 (4)	0
PD	9 (33)	2 (33)
HD home and hospital	2 (7)	1 (17)
PD and HD	1 (4)	0

*tx* transplantation, *HD* haemodialysis, *PD* peritoneal dialysis

**Table 3:** Participant characteristics; health-care personnel caring for patients during the wait for transplantation and health-care personnel with transplant specific expertise

	**HCP CKD-care (*n* = 20)**	**HCP tx spec. (*n* = 14)**
Age year, Mean (SD)	42 (10)	52 (15)
Gender, female n (%)	19 (95)	12 (86)
Years in HC profession, Mean (SD)	18 (10)	28 (13)
Years in renal care, Mean (SD)	15 (10)	22 (13)

HCP CKD-care—health-care personnel working with patients with chronic kidney disease; HCP tx spec—health-care personnel with transplant specific expertise

Health-care personnel in the care of patients awaiting kidney transplantation included hemodialysis nurses, peritoneal dialysis nurses, pre-dialysis nurses, nephrologists in nephrology departments and renal social workers. The mean age was 42 years and 95 per cent were female. Health-care personnel with kidney transplant specific experience included physiotherapists with transplant expertise, transplant patient coordinators, transplant surgeons, transplant nephrologists, nurses working in renal transplantation in-patient care and nurses working with transplant out-patient follow-up. The mean age was 52 years and 86 per cent were women.

#### Quantitative results

All items, for which we asked participants to rate the importance, had a median rating of 4 or 5, hence consensus was reached in all items in round 1, see Table 4.

**Table 4:** Items and ratings in round 1

**Items Round 1** -completed by 67 participants	**Ratings Round 1** *1 not important- 5 very important* Median (Q1, Q3)
*Week 1—Introduction*	
1. How important do you think it is for this support and education program that the person gets to write about their experience of their kidney disease before setting goals for the program?	5 (4, 5)
2. How important do you think it is for this support and education program to encourage regular physical activity?	5 (4, 5)
*Week 2 – Living with CKD and waiting for transplantation*	
3. Information about the kidneys	5 (4, 5)
4. Information about kidney disease and kidney failure	5 (4, 5)
5. Information about dialysis	5 (4, 5)
6. Information about what life with dialysis may be like, regarding relations and sexuality	5 (4, 5)
7. Information about what life with dialysis may be like, regarding diet	5 (4, 5)
8. Information about what life with dialysis may be like, regarding activities	5 (4, 5)
9. Information about access to advice and support for those with kidney disease	5 (4, 5)
10. Information about peritoneal dialysis as a treatment	5 (4, 5)
11. Information about hemodialysis as a treatment	5 (4, 5)
12. Information that kidney disease can cause uncertainty in life—about fear and anxiety	5 (4, 5)
13. Information that significant others often also experience fear and anxiety	5 (4, 5)
14. Information about how work and sick leave can affect a person during the wait for kidney transplantation and how soon a return to work is usually relevant after kidney transplantation	5 (4, 5)
15. Information about what is required to be able to have a kidney transplant	5 (5, 5)
16. Differences in kidney transplantation with a living/deceased donor	4 (4, 5)
17. Information about transplant compatibility, blood types and tissue type	4 (4, 5)
18. Information about the Scandinavian kidney exchange program	4 (3, 5)
19. Information about what to expect from a transplanted kidney	5 (5, 5)
20. Information about how the waiting list works	5 (5, 5)
21. Information about temporary deactivation on the waiting list for, for example, illness or travel. How and why	5 (4, 5)
22. Information that examinations performed as part of the transplant investigation may need to be renewed during the waiting period, and that in the event of deteriorating health conditions, new decisions may need to be made about transplantation	5 (4, 5)
23. Information about mental stress during the waiting period	5 (4, 5)
24. Preparations for the trip to the transplant center	5 (4, 5)
25. Packing list—what to take with you when it is time for the transplant	4 (3, 5)
26. Information about self-care for persons who want to have a kidney transplant—Why different self-care activities are important for persons who are waiting for a transplant	5 (4, 5)
27. What happens when the call for a kidney transplant comes	5 (5, 5)
28. What will happen at the transplant center; before, during and after transplantation	5 (4, 5)
29. Psychological reactions that are common after transplantation	5 (4, 5)
30. Follow-up after transplantation	5 (4, 5)
31. Basic information about medication used after transplantation and their most common side-effects	5 (5, 5)
32. How one may expect to feel after the transplant	5 (5, 5)
33. Basic information about complications after kidney transplantation	5 (4, 5)
34. Taking your medication correctly; why it is important and tips on how to facilitate it	5 (5, 5)
35. Tobacco and how it can affect the body after transplantation	5 (4, 5)
36. Physical activity and how it affects the body after the transplant	5 (4, 5)
37. Dietary advice	5 (4, 5)
38. Food and hand hygiene	5 (4, 5)
39. Sun protection	5 (5, 5)
40. To be observant of your body for any symptoms of side effects or complications	5 (5, 5)
41. About relations, sexuality, contraception and pregnancy after transplantation	5 (4, 5)
42. Information on working after transplantation	5 (4, 5)
*Week 3 – Acceptance*	
43. How important do you think Acceptance training is for the support and training program?	5 (4, 5)
44. How important is it that examples are given that make it easier for the participant to link the exercises to their own situation?	5 (4, 5)
45. How important is it in a chapter on acceptance to see and hear a patient talk about his or her experience of waiting for a transplant?	5 (4, 5)
*Week 4 – Problem solving*	
46. How important do you think Problem Solving is for the support and training program?	5 (4, 5)
47. How important is it that examples of problem solving are about kidney failure and waiting for a kidney transplant?	5 (4, 5)
Week 5, 6, 7 – Not putting life on hold (calendar therapy, behavioural activation)	
48. How important do you think it is that this part of the program is spread over three weeks?	4 (3, 5)
49. How important do you think it is to learn to take advantage of the time while waiting for a transplant, despite dialysis and illness?	5 (4, 5)
50. How important do you think learning to break negative circles and dwelling are for the support and education program?	5 (5, 5)
51. How important do you think calendar therapy is for the support and education program?	4 (4, 5)
Week 8 – completion	
52. How important do you think it is to create an individual action plan to return to regularly during the remainder of the waiting period for transplantation, or when needed?	5 (4, 5)
Program design	
53. What do you think about the length of the program of 8 weeks?	4 (3, 5)
54. How valuable do you think it is for program participants to take part of other patients' experiences of kidney disease, dialysis and transplantation, in form of quotes?	5 (4, 5)
55. How important do you think it is to see patients tell about their experiences in the form of films?	5 (4, 5)
56. How important is it that parts of the program information is told in form of films?	4 (4, 5)
57. How important do you think it is to have a lot of pictures and figures in the program?	4 (3, 5)
58. How important do you think it is that the participants are asked to submit their homework and any questions to their contact person every week, and each week they receive feedback on what they submit?	4 (3, 5)
59. Do you think patients should be offered this type of support and education? Yes/No	Yes 100%

*Q1 *quartile 1, *Q3 *quartile 3

There were significant differences between the groups regarding the items Encourage physical activity (*p* = 0.013), Weekly assignments and feedback (*p* = 0.017) and Importance of figures and pictures in the program (*p* = 0.003) which were ranked higher by healthcare personnel compared to patients and significant others jointly. Information regarding kidneys (*p* = 0.02), Information about chronic kidney disease (*p* = 0.045), and Information about transplantation compatibility (*p* = 0.009) were all ranked higher by patients and significant others compared to health-care personnel. Among patients there was a significant positive association between waiting time for transplantation and preferring behavioral activation spanning over three weeks (*R* = 0.468, *p* = 0.014). There was also a significant positive association between waiting time and preference for taking part of other patients' experiences in form of quotes (*R* = 0.545, *p* = 0.003). No other content or designs items had significant associations with patient age or waiting time. In a yes/no question, all participants (100%) marked yes, indicating that the program should be offered to patients awaiting kidney transplantation.

#### Qualitative findings

A total of 614 free-text comments were provided, i.e. 9.2 comments per participant.

In the category *Program content* there were suggestions to add information about life as a primary caregiver to young children when you have chronic kidney disease, if and how to inform children of the parent´s illness and treatment. There were also suggestions about expanding the transplantation specific information.

In the category *Program layout and design* feedback encompassed that a substantial amount of crucial information was present in the program prototype. Additionally, recommendations were made to tailor the content to individual requirements and preferences. This could be achieved by making certain information available based on individual needs and wishes.

The category *Implementation aspects* contained suggestions including expansion of the program to make more room for transplantation specific information. There were also suggestions regarding competencies of the support person of the program, including extensive knowledge about kidney disease, dialysis and kidney transplantation as well as long experience of working in renal care. Desired characteristics of the support person were professionality, empathy, being a good listener and ability to motivate and support persons in reaching set goals. The support person was suggested to be the same throughout the program to enable building trust, and to provide solely written feedback or a combination of written and oral feedback.

### Results round 2

#### Participants

Of the 67 participants who completed round 1 and hence were invited to participate in round 2, 53 completed round 2. Compared with those who dropped out, those who completed the second round (*n* = 53) did not significantly differ regarding mean age (47 vs. 52 years, *p* = 0.21), gender (females 71% vs. 74%, *p* = 0.87) and group (*p* = 0.44).

#### Quantitative results

Based on comments in round 1, topics that could be expanded on in the program were suggested and constituted items for rating in round 2. Items were rated on a scale 1–5 with median ratings 4–5, see Table 5, hence consensus was reached on all items in round 2 with no statistical differences between the groups.

**Table 5:** Items and ratings in round 2

**Items Round 2,** -completed by 53 participants	**Ratings Round 2, 4 new items** *1 not important- 5 very important* Median (Q1, Q3)
1. How important do you think it is that the program informs about what it may be like to have young children as relatives and how to inform them of one´s illness and treatment?	5 (4, 5)
2. How important do you think it is that the program puts more focus on information about transplantation and life as a transplant recipient?	5 (4, 5)
3. How important do you think it is that the program is extended by one week to a total of 9 weeks, to provide more time for the information previously contained in week 2, (i.e. information about kidney disease, dialysis, transplantation and life while waiting for kidney transplantation, work, economy)?	4 (3.5, 5)
4. How important do you think it is that the program provides the opportunity to choose how much you want to read about certain topics? For example, to get brief texts about different forms of dialysis and be able to choose to read more about, for example, peritoneal dialysis by clicking on a button for "Do you want to know more about peritoneal dialysis, click here"	5 (4, 5)

#### Qualitative findings

Fewer free-text comments were provided in round 2, but 90 per cent of the comments provided were full sentences. Most comments expressed encouragement for the intervention development, some explained reasons for ratings of certain items and stressing importance of realistic yet well-balanced information in the program. No participants reported having problems understanding the questionnaires.

## Discussion

To our knowledge, this is one of the first studies involving heterogenous experts in the development of an internet-based support and education intervention. This study focused on patients awaiting kidney transplantation from deceased donors with the aim to further develop and refine program content and design. Using a modified Delphi-approach, patients who had received a kidney transplant from a deceased donor within the last 2 years, their significant others, health-care personnel working in renal care and kidney transplant specialist personnel were asked to take part in two to three rounds via a web-questionnaire. Consensus was reached on all items rated in round 1, where all items reached median ratings of 4–5, hence considered important for the planned support and education program. Early consensus allowed utilization of the second round for further evaluation of qualitative findings, where four new content and design items were rated, on which consensus was reached. Thus, in alignment with the MRC/NIHR framework [5] we have in an iterative process further refined our intervention resulting in the following adaptations. Information about life with young children as relatives when you have chronic kidney disease was added. The kidney transplantation specific information was expanded and to allow this, the program was extended by one week. Lastly, the program was individualized by making certain educational texts available and optional on different levels.

Although there was an overall consensus for all items, there were some minor and expected differences found between participant groups. Patients and significant others rated the importance of education on kidneys, chronic kidney disease and transplantation compatibility higher, indicating that they want more education than health-care personnel believe necessary. On the other hand, health-care personnel rated the importance of encouraging physical activity higher than the patients did. It may be important to consider what these differences represent when developing an intervention. Our results cannot explain the reasons behind participants' specific ratings. However, we believe that considering patients' expressed need for more information, it is logical to assume that they require detailed information about the mechanisms and impacts of specific aspects, like physical activity. This need for understanding is essential for them to grasp the significance of these factors in their context. We therefore, as part of the refinement of our education and support program, find it important to incorporate information that clearly explains why self-care activities are important for the patients themselves, as this may help motivate the patients to actually perform self-care activities such as physical activity [21].

Despite the education and support program prototype largely being inspired by CBT, and only one week focusing education on kidney disease and treatment, participants suggested that the support person should have extensive knowledge and experience of chronic kidney disease, dialysis and transplantation. These suggestions are supported by previous research, where health-care professionals, such as nurses, with clinical experience of certain medical disease, after brief CBT-training have delivered CBT for their patients with good results [22, 23].

We suggest that a guided internet-based support and education program for patients awaiting renal transplantation with deceased donors may comprise content as per our prototype (Table 1), with one additional week to allow greater focus on transplantation education guided by a support person with clinical experience of renal and transplantation care. Furthermore, we advise that information regarding young children as relative is included. We propose that each module contains text which may be individualized by making certain information available based on individual needs and wishes, illustrations, short videos, weekly homework assignments and a clearly motivated prompt for physical activity. Using visual aids like figures, pictures, and short videos may also be a way to enhance the learning experience for individuals across various educational levels as well as for patients whose learning is impacted on by uremic symptoms.

Digital literacy is a challenge that has to be considered when developing an internet-based support and education program. While internet usage in Sweden is high (90 percent are daily internet users) [24] within the target population, we believe that an internet support and education program will be suitable for many, but not all. This will, in turn, free up face-to-face resources for those individuals who, for various reasons, may not benefit from internet intervention.

### Strengths and limitations

Among the limitations for the Delphi method is that reliability increases with the size of the group and number of questionnaire rounds but response rates tend to decrease with the number of rounds, hence decreasing reliability and making it difficult to reach a meaningful result. Also, coordinating large groups and many rounds can be complicated, time consuming and hence costly [17, 18]. It is also known that patients and health care personnel often differ in opinions regarding healthcare aspects, such as found by Bortoli et al. [25], which may prolong the consensus process, but on the other hand may enrich the results of the Delphi procedure [18]. Bearing this in mind, we planned to perform telephone interviews with those who expressed deviating opinions, in case of consensus not being reached after round two, to clarify reasons for disagreements and achieve greater understanding of opinions. Feedback with results from round two and interview findings would be sent to the expert panel for a third and final round, but these steps were not required in this study. Surprisingly, despite heterogeneity, consensus was reached on all items being important for the program, in the first round. One reason for early consensus may be that the patient group in question has great education and support needs and scarce support resources available. Early consensus on all suggested items may also be associated with the program prototype, which had been thoroughly developed based on previous research on the topic, patient information from Sweden’s four transplantation centers, the research group’s considerable experience of ICBT in chronic conditions as well as the first author´s extensive clinical experience of working with this group of patients and their complex needs, which is a strength. It is, however, a possible limitation that the participants, although having different backgrounds, expertise and experience may not know much about CBT. Participants may not have suggested changes to the support aspects because they simply did not have any ideas of what may be changed and how. There was a high percentage of whole sentences in written feedback in both questionnaire rounds, which may indicate a solid level of engagement in the discussion and serve as a quality measure [26]. As in all studies using voluntary participation a selection bias arises. In this study participants may be inclined towards patient education, possibly predisposing the study towards favorable outcomes. However, it is important to note that the health-care personnel who took part in the study were likely to have encountered patients who exhibited a lack of interest or prior knowledge in the subject matter. Similarly, the participating patients had the experience of awaiting transplantation, which might not necessarily have been accompanied by a proactive interest during the waiting time. However, the hindsight gained from this experience could have led them to recognize certain informational needs that were previously not apparent.

Recruitment through clinical managers proved difficult during this time of pandemic, why we expanded recruitment to social media for patients and interest organizations for health care personnel. Doing so we enabled representation of all parts of the country, as well as small and large hospitals and Sweden’s all four transplant centers, which we consider a strength of our study. However, when opening up for self-registration for participation we did in some ways lose control of the participants, who may register untrue data about themselves in order to qualify for participation. Four participants who completed round 1, of which three also completed round 2, did not fulfill the inclusion criteria, having been transplanted between 9 months to 16 years longer than prescribed for inclusion. Upon discussion within the research team we decided to include this data, as these persons, despite many years after transplantation had taken time for participation, finding it unethical to exclude them. Also, these persons´ data did not in any way alter the outcomes of the study.

Our study was conducted in Sweden, where healthcare is nearly free and the social insurance system is well-established. We believe that our results hold relevance in other countries as well, but it is important to consider the variations in healthcare and social insurance systems that operate differently in each country and similar to all support interventions, cultural adaptation needs to be considered before application to a new cultural context [27].

## Conclusions

There was consensus among a heterogenous group of experts regarding suggested educational and psychological support content and design aspects, and additional content and design aspects were identified for an internet-based support and education program for patients awaiting kidney transplantation from deceased donors. We found the Delphi method useful for further developing and refining program content and design.

## Data Availability

The datasets used and analysed during the current study are available from the corresponding author on reasonable request.

## References

[1] Gibbons A, Bayfield J, Bradley C, Cinnirella M, Draper H, Johnson RJ, Oniscu GC, Metcalfe W, Forsythe JLR, Ravanan R (2021). Changes in quality of life (QoL) and other patient-reported outcome measures (PROMs) in living-donor and deceased-donor kidney transplant recipients and those awaiting transplantation in the UK ATTOM programme: a longitudinal cohort questionnaire survey with additional qualitative interviews. BMJ Open.

[2] Koons B, Smeltzer SC (2018). The patient experience of waiting on the deceased donor kidney transplant list while receiving dialysis. Nephrol Nurs J.

[3] Nilsson K, Westas M, Andersson G, Johansson P, Lundgren J (2022). Waiting for kidney transplantation from deceased donors: experiences and support needs during the waiting time -a qualitative study. Patient Educ Couns.

[4] Rosaasen N, Mainra R, Shoker A, Wilson J, Blackburn D, Mansell H (2017). Education before kidney transplantation: what do patients need to know?. Prog Transplant.

[5] Skivington K, Matthews L, Simpson SA, Craig P, Baird J, Blazeby JM, Boyd KA, Craig N, French DP, McIntosh E (2021). A new framework for developing and evaluating complex interventions: update of medical research council guidance. BMJ.

[6] Johansson P, Westas M, Andersson G, Alehagen U, Broström A, Jaarsma T, Mourad G, Lundgren J (2019). An internet-based cognitive behavioral therapy program adapted to patients with cardiovascular disease and depression: randomized controlled trial. JMIR Ment Health.

[7] Beck AT, Dozois DJ (2011). Cognitive therapy: current status and future directions. Annu Rev Med.

[8] Hayes S, Pierson H: Acceptance and commitment therapy. In: Encyclopedia of cognitive behavior therapy. edn.: Springer; 2005: 1–4.

[9] Karyotaki E, Efthimiou O, Miguel C, Bermpohl FMG, Furukawa TA, Cuijpers P, Riper H, Patel V, Mira A, Gemmil AW (2021). Internet-based cognitive behavioral therapy for depression: a systematic review and individual patient data network meta-analysis. JAMA Psychiat.

[10] Veehof MM, Trompetter HR, Bohlmeijer ET, Schreurs KM (2016). Acceptance- and mindfulness-based interventions for the treatment of chronic pain: a meta-analytic review. Cogn Behav Ther.

[11] Bonnert M, Hedman E, Melin B, Ljótsson B, Lalouni M, Engelbrektsson J, Lenhard F, Vigerland S, Serlachius E, Olén O (2017). Internet-delivered cognitive behavior therapy for adolescents with irritable bowel syndrome: a randomized controlled trial. American J Gastroenterol.

[12] Li Y, Storch EA, Ferguson S, Li L, Buys N, Sun J (2022). The efficacy of cognitive behavioral therapy-based intervention on patients with diabetes: a meta-analysis. Diabetes Res Clin Pract.

[13] Rickardsson J, Gentili C, Holmstrom L, Zetterqvist V, Andersson E, Persson J, Lekander M, Ljotsson B, Wicksell RK (2021). Internet-delivered acceptance and commitment therapy as microlearning for chronic pain: a randomized controlled trial with 1-year follow-up. European J Pain (London, England).

[14] Cuijpers P, Pineda BS, Quero S, Karyotaki E, Struijs SY, Figueroa CA, Llamas JA, Furukawa TA, Muñoz RF (2021). Psychological interventions to prevent the onset of depressive disorders: a meta-analysis of randomized controlled trials. Clin Psychol Rev.

[15] Schmitt JC, Valiente RM, García-Escalera J, Arnáez S, Espinosa V, Sandín B, Chorot P (2022). Prevention of depression and anxiety in subclinical adolescents: effects of a transdiagnostic internet-delivered CBT program. Intern J Environ Res Pub Health.

[16] Humphrey-Murto S, Varpio L, Wood TJ, Gonsalves C, Ufholz LA, Mascioli K, Wang C, Foth T (2017). The use of the delphi and other consensus group methods in medical education research: a review. Acad Med.

[17] Fink A, Kosecoff J, Chassin M, Brook RH (1984). Consensus methods: characteristics and guidelines for use. Am J Public Health.

[18] Boulkedid R, Abdoul H, Loustau M, Sibony O, Alberti C (2011). Using and reporting the Delphi method for selecting healthcare quality indicators: a systematic review. PLoS ONE.

[19] von Elm E, Altman DG, Egger M, Pocock SJ, Gøtzsche PC, Vandenbroucke JP (2007). Strengthening the Reporting of Observational Studies in Epidemiology (STROBE) statement: guidelines for reporting observational studies. BMJ.

[20] Lincoln YS, Guba EG (1985). Naturalistic inquiry.

[21] Riegel B, Jaarsma T, Lee CS, Strömberg A (2019). Integrating symptoms into the middle-range theory of self-care of chronic illness. ANS Adv Nurs Sci.

[22] Tyrer H, Tyrer P, Lisseman-Stones Y, McAllister S, Cooper S, Salkovskis P, Crawford MJ, Dupont S, Green J, Murphy D (2015). Therapist differences in a randomised trial of the outcome of cognitive behaviour therapy for health anxiety in medical patients. Int J Nurs Stud.

[23] Westas M, Mourad G, Lundgren J, Johansson P, Andersson G, Neher M (2022). The experience of participating in an internet-based cognitive behavioral therapy program among patients with cardiovascular disease and depression: a qualitative interview study. BMC Psychiatry.

[24] The Swedish Internet Foundation: Svenskarna och internet [The Swedes and the Internet]. 2022.

[25] Bortoli A, Daperno M, Kohn A, Politi P, Marconi S, Monterubbianesi R, Castiglione F, Corbellini A, Merli M, Casella G (2014). Patient and physician views on the quality of care in inflammatory bowel disease: results from SOLUTION-1, a prospective IG-IBD study. J Crohns Colitis.

[26] Beiderbeck D, Frevel N, von der Gracht HA, Schmidt SL, Schweitzer VM (2021). Preparing, conducting, and analyzing Delphi surveys: cross-disciplinary practices, new directions, and advancements. MethodsX.

[27] Chu J, Leino A (2017). Advancement in the maturing science of cultural adaptations of evidence-based interventions. J Consult Clin Psychol.

[28] World Medical Association Declaration of Helsinki (2013). ethical principles for medical research involving human subjects. JAMA.

